# Emotion Regulation Difficulties as a Statistical Mediator of the Association Between Alexithymia and Coping Strategies in Adolescents

**DOI:** 10.3390/children13040462

**Published:** 2026-03-27

**Authors:** Yurdagül Selvi, Nuray Şimşek

**Affiliations:** 1 Institute of Health Sciences, Erciyes University, 38039 Kayseri, Turkey; 2 Faculty of Health Sciences, Department of Nursing, Nuh Naci Yazgan University, 38170 Kayseri, Turkey; 3 Faculty of Health Sciences, Department of Nursing, Erciyes University, 38039 Kayseri, Turkey

**Keywords:** alexithymia, emotion regulation, coping behavior, adolescent, psychological adaptation

## Abstract

**Background**: Adolescence is a sensitive developmental period marked by heightened emotional reactivity and increasing demands on emotion recognition and regulation. Although alexithymia has been associated with less adaptive and avoidant coping tendencies in adolescents, most prior research has relied on descriptive or bivariate approaches, leaving the underlying processes and model-based pathways insufficiently clarified. In particular, the explanatory role of difficulties in emotion regulation in the association between alexithymia and coping strategies remains underexplored. This study aimed to address this gap by examining whether difficulties in emotion regulation mediate the relationship between alexithymia and coping strategies in adolescents. **Methods**: In this cross-sectional study, 1415 adolescents (13–17 years) from public high schools in Central Anatolia, Türkiye, completed the Toronto Alexithymia Scale (TAS-20), the Difficulties in Emotion Regulation Scale (DERS-16), and the Coping Strategies Indicator (CSI). Pearson correlations were calculated. Mediation analyses were conducted using PROCESS Macro (Model 4) with 5000 bootstrap samples, adjusting for age, gender, academic achievement, and family type. **Results**: Alexithymia was moderately associated with emotion regulation difficulties (r = 0.49, *p* < 0.001). Mediation analyses revealed significant indirect effects for seeking social support (B = −0.068, 95% CI [−0.087, −0.051]) and problem solving (B = −0.067, 95% CI [−0.086, −0.049]), with direct effects remaining significant, indicating inconsistent (competitive) mediation patterns. For avoidance coping, the indirect effect was significant (B = −0.072, 95% CI [−0.090, −0.055]), whereas the direct effect became non-significant, consistent with an indirect-only mediation pattern. Correlations involving coping outcomes were small in magnitude. According to Cohen’s criteria, the association between alexithymia and emotion regulation difficulties was moderate in magnitude, whereas correlations involving coping outcomes were small. **Conclusions**: Difficulties in emotion regulation emerged as a statistical mediator within the proposed model, demonstrating systematic associations between alexithymia and distinct coping patterns in adolescents. These findings underscore the relevance of emotion regulation–focused prevention and intervention efforts in school settings. By examining multiple coping outcomes simultaneously within a covariate-adjusted mediation framework in a large community adolescent sample, this study offers an integrative, model-based perspective on how alexithymic traits are linked to coping through regulatory difficulties.

## 1. Introduction

The ability to recognize, identify, and regulate emotions is a fundamental determinant of psychological well-being across the lifespan [1]. Adolescence represents a particularly critical developmental period, during which rapid biological, cognitive, and social changes place increased demands on emotional processing and regulation skills [1]. During this stage, many adolescents experience difficulties in recognizing, understanding, and managing their emotional experiences, which may increase vulnerability to maladaptive coping behaviors and psychological distress [2].

Alexithymia refers to a multidimensional construct characterized by difficulties in identifying and describing emotions, as well as a tendency toward externally oriented thinking [3]. Accumulating evidence indicates that adolescents with elevated alexithymic traits are less likely to engage in adaptive coping strategies, such as problem solving and seeking social support, and more likely to rely on avoidant or disengagement-oriented coping strategies [4]. Although avoidance is often associated with poorer long-term adjustment when used rigidly or chronically, it can also be context-dependent and may serve short-term emotion regulation or strategic disengagement from uncontrollable stressors, particularly in early adolescence. However, the mechanisms through which alexithymia influences coping behaviors during adolescence remain insufficiently understood.

Importantly, alexithymia has also been conceptualized within a developmental and relational framework. Emerging evidence suggests that emotionally invalidating, rejecting, or abusive caregiving environments may contribute to the development of alexithymic traits by limiting opportunities for emotional labeling, validation, and reflective dialogue about internal states [5]. In this context, chronic disruptions in parent–child emotional communication may hinder the acquisition of emotional awareness and differentiation skills. Beyond its developmental origins, alexithymia has been consistently associated with broader psychological and relational difficulties, including increased internalizing symptoms, impaired interpersonal functioning, reduced empathy, and lower relationship quality. These findings underscore that alexithymia is not merely an individual emotional deficit but may reflect disruptions in socio-emotional development with meaningful implications for adolescent psychological adjustment [5].

Emotion regulation represents a core psychological process that shapes how individuals respond to emotional experiences and cope with stressors [6]. Emotion regulation broadly refers to the processes by which individuals monitor, evaluate, and modify the intensity, duration, and expression of emotional responses in order to achieve situational goals or maintain psychological equilibrium. These processes may involve cognitive, behavioral, and interpersonal strategies that influence how emotions are experienced and expressed. Within this framework, regulation strategies are often categorized as adaptive or maladaptive depending on their functional consequences. Adaptive strategies—such as cognitive reappraisal, problem solving, and seeking social support—facilitate constructive engagement with stressors, promote emotional clarity, and are generally associated with better psychological and interpersonal outcomes. In contrast, maladaptive strategies—such as avoidance, suppression, and emotional disengagement—may reduce distress in the short term but are frequently linked to poorer long-term adjustment and increased psychological vulnerability [6].

Deficits in emotion regulation are particularly salient during adolescence due to ongoing neurodevelopmental changes, heightened emotional reactivity, and increasing social and academic demands [7]. Previous research has consistently shown that adolescents with greater difficulties in emotion regulation tend to employ fewer adaptive coping strategies and exhibit a higher reliance on maladaptive coping patterns [6]. Moreover, individuals with alexithymic traits have been shown to demonstrate pronounced impairments in emotion regulation, suggesting a close and theoretically meaningful link between these constructs [4].

Although alexithymia, emotion regulation, and coping strategies have each been extensively examined, prior research has often relied on bivariate associations or single-outcome models, leaving the underlying processes linking alexithymia to coping insufficiently clarified—particularly during adolescence [6]. Fewer studies have tested these constructs simultaneously within a unified, covariate-adjusted mediation framework that allows comparison across multiple coping strategies within the same analytical model [4,8]. To our knowledge, limited research has examined whether emotion regulation difficulties may be differentially associated with adaptive versus maladaptive coping strategies within the same mediation framework. In particular, the possibility that adaptive coping strategies may exhibit indirect-only patterns, whereas avoidance coping may retain both direct and indirect pathways, has not been sufficiently explored within a single covariate-adjusted framework. Building on this analytical perspective, the present study adopts a model-based approach to examine emotion regulation difficulties as a statistical mediator within a unified explanatory model linking alexithymia to multiple coping strategies in a large adolescent sample, while adjusting for key demographic factors. By delineating strategy-specific mediation patterns within the same framework, this study aims to contribute to a more differentiated and clinically informative understanding of adolescent emotional adjustment.

In addition to testing the mediation model, demographic variables were examined both descriptively and as statistical controls to strengthen the interpretability of the findings. Gender has been consistently linked to differences in emotional awareness, emotion regulation strategies, and coping patterns during adolescence, with males tending to report higher alexithymia in certain dimensions and females more frequently reporting internalizing symptoms and emotion-focused coping. Accordingly, gender was considered a theoretically relevant contextual factor in the present study.

Academic achievement was also included, as emotional processing, regulation capacities, and coping strategies are closely related to academic engagement and school adjustment. Difficulties in identifying and regulating emotions may hinder adaptive responses to academic stressors, highlighting the broader functional relevance of these constructs.

Based on prior theoretical and empirical evidence, we expected higher alexithymia to be associated with lower adaptive coping and higher maladaptive coping. Greater emotion regulation difficulties were similarly expected to relate to maladaptive coping and to mediate the association between alexithymia and coping strategies. Specifically, adaptive coping was anticipated to demonstrate primarily indirect effects through emotion regulation difficulties, whereas maladaptive coping—particularly avoidance—was expected to retain both direct and indirect associations with alexithymia.

### Research Questions

1. What are the levels of alexithymia, difficulties in emotion regulation, and coping strategies among adolescents?
2. How are alexithymia, difficulties in emotion regulation, and coping strategies interrelated in adolescents?
3. Do difficulties in emotion regulation mediate the relationship between alexithymia and coping strategies in adolescents?
4. Do demographic characteristics, including age, gender, academic achievement, and family type, influence alexithymia, difficulties in emotion regulation, and coping strategies?

## 2. Materials and Methods

### 2.1. Study Design

This study employed a cross-sectional, correlational research design. Mediation effects were examined using regression-based path analysis implemented through Hayes’ PROCESS macro (Model 4), with 5000 bias-corrected bootstrap samples to generate confidence intervals for indirect effects. In addition to examining bivariate associations, the study tested whether difficulties in emotion regulation statistically mediated the associations between alexithymia and multiple coping strategies [9].

### 2.2. Participants

The study population consisted of 2909 students enrolled in public high schools located in the central districts of a province in the Central Anatolia Region of Türkiye. Data were collected from students attending 3 public middle and high schools. Schools were selected following administrative approval. A school-based, non-probability convenience sampling strategy was employed. Within participating schools, students present in selected classes during scheduled data collection sessions were screened for eligibility and invited to participate voluntarily.

Eligibility criteria were: (i) being between 13 and 17 years of age, (ii) current enrollment in middle and high school, and (iii) provision of written informed consent by both adolescents and their parents. Students who did not meet the predefined age criterion or whose parents did not provide written consent were not included in the study and were not administered the questionnaires.

Of the 2909 students enrolled in the participating schools, 233 were excluded because written parental consent was not obtained. An additional 1189 students did not meet the predefined age criterion and were therefore not eligible for inclusion. The remaining students met eligibility requirements and were invited to participate. Among those eligible and consented, 1487 completed the questionnaires. Seventy-two cases were excluded due to incomplete or substantially missing responses. The final analytic sample consisted of 1415 adolescents. Data collection was conducted between October 2022 and January 2023.

### 2.3. Study Variables

Predictor variable (X): Alexithymia (TAS-20 total score)

Mediator variable (M): Difficulties in emotion regulation (DERS-16 total score)

Outcome variables (Y): Coping strategies (CSI subscales: seeking social support, problem solving, avoidance)

Covariates: Age, gender, academic achievement, and family type

### 2.4. Data Collection Instruments

Data were collected using the Personal Information Form, Toronto Alexithymia Scale, Difficulties in Emotion Regulation Scale, and Coping Strategies Indicator.

#### 2.4.1. Personal Information Form

The Personal Information Form was developed by the researchers based on the relevant literature to assess adolescents’ sociodemographic characteristics. The form included items related to age, family type, grade level, and academic achievement.

#### 2.4.2. Toronto Alexithymia Scale (TAS-20)

The Toronto Alexithymia Scale, developed by Bagby, Parker, and Taylor (1994) [10], consists of 20 items and three subscales: (1) Difficulty Identifying Feelings, (2) Difficulty Describing Feelings, (3) Externally Oriented Thinking. Higher scores on the TAS-20 indicate greater levels of alexithymia, reflecting increased difficulty in recognizing, describing, and cognitively processing emotional experiences.

The Cronbach’s alpha coefficients for the original scale were reported as 0.78, 0.75, and 0.66, respectively, with an overall reliability coefficient of 0.81. The Turkish validity and reliability study was conducted by Güleç et al. (2009) [11].

In the present study, the Cronbach’s alpha coefficient for the total TAS-20 score was 0.77, and McDonald’s omega (ω) was 0.79, indicating acceptable internal consistency. The reliability coefficients for the subscales were 0.82 for difficulty identifying feelings, 0.74 for difficulty describing feelings, and 0.71 for externally oriented thinking.

#### 2.4.3. Difficulties in Emotion Regulation Scale (DERS-16)

The DERS-16, developed by Bjureberg et al. (2016) [12], is used to assess difficulties in emotion regulation. Higher scores on the DERS-16 indicate greater difficulties in emotion regulation, reflecting poorer emotional awareness, acceptance, and regulation abilities. The original scale has a total Cronbach’s alpha value of 0.92 and a test–retest reliability of 0.85. The Turkish validity and reliability study was conducted in 2019 [13]. In this study, the Cronbach’s alpha coefficient of the DERS-16 was 0.89, and McDonald’s omega (ω) was 0.94, indicating excellent internal consistency.

#### 2.4.4. Coping Strategies Indicator (CSI)

The Coping Strategies Indicator was developed by Amirkhan (1990) [14] and adapted into Turkish by Aysan (2003) [15]. The scale consists of three subscales: Seeking Social Support, Problem Solving and Avoidance. Higher scores on Seeking Social Support and Problem Solving indicate greater use of generally adaptive coping strategies. Avoidance is often associated with less adaptive coping outcomes, particularly in the context of persistent stress; however, its adaptiveness may vary depending on situational and developmental factors.

The overall reliability coefficient of the scale is 0.92. Cronbach’s alpha coefficients for the subscales were reported as 0.86 for seeking social support, 0.83 for problem solving, and 0.70 for avoidance [14].

In the present study, the Cronbach’s alpha coefficient of the CSI was 0.96, while the coefficients for the subscales were 0.84, 0.79, and 0.75, respectively. In addition, McDonald’s omega (ω) for the total CSI score was 0.85, providing further evidence of satisfactory internal consistency.

### 2.5. Data Collection Procedure

Students and their parents were informed about the study objectives and procedures prior to participation. Written informed consent was obtained from both adolescents and their parents. Data were collected through face-to-face administration of questionnaires by the researcher, with the support of school counselors, during scheduled school hours. Completion of the questionnaires took approximately 30 min.

### 2.6. Statistical Analysis

Data were analyzed using IBM SPSS Statistics version 22.0. Descriptive statistics were reported as frequency (n), percentage (%), mean (M), standard deviation (SD), median, and minimum–maximum values, as appropriate. The normality of continuous variables was initially assessed using the Shapiro–Wilk test. However, given the large sample size (N = 1415), formal normality tests were interpreted cautiously, as even trivial deviations may yield statistically significant results in large samples. Therefore, normality assumptions were evaluated using a combination of statistical indicators (skewness and kurtosis values) and visual inspection of histograms. In line with conventional thresholds, distributions were considered approximately normal when skewness and kurtosis values fell within acceptable ranges.

Group comparisons between two independent groups were conducted using the Independent Samples *t*-test for approximately normally distributed variables. Comparisons among three or more groups were performed using one-way analysis of variance (ANOVA). Given the large sample size, ANOVA was considered robust to moderate deviations from normality. When significant omnibus effects were observed, post hoc comparisons were conducted using Tukey’s HSD test to identify specific group differences. Pearson correlation coefficients were calculated to examine associations among alexithymia, difficulties in emotion regulation, coping strategies, and age. To test the proposed mediation models, regression-based mediation analyses were conducted using Hayes’ PROCESS macro for SPSS (Model 4). Total (c path), direct (c′ path), and indirect (a × b) effects were estimated. Indirect effects were evaluated using 5000 bias-corrected bootstrap samples with 95% confidence intervals. Age, gender, academic achievement, and family type were included as covariates in all mediation models. Indirect effects were considered statistically significant when the 95% bootstrap confidence interval did not include zero. A two-tailed *p*-value < 0.05 was considered statistically significant for all analyses.

## 3. Results

The mean age of the adolescents included in the study was 14.92 ± 0.94 years. Of the participants, 59% were female, 37.3% were ninth-grade students, 82.3% lived in nuclear families, 73.9% reported a moderate family income, and 65.6% indicated a moderate level of academic achievement.

Descriptive statistics for alexithymia, difficulties in emotion regulation, and coping strategies are presented in Table 1. The mean total score of the Toronto Alexithymia Scale (TAS-20) was 55.06 (SD = 10.21), indicating moderate levels of alexithymia in the sample. The mean total score of the Difficulties in Emotion Regulation Scale (DERS-16) was 41.30 (SD = 15.31), reflecting a wide range of emotion regulation difficulties among adolescents. Regarding coping strategies, mean scores for the Coping Strategies Indicator subscales were 22.00 (SD = 5.33) for seeking social support, 20.18 (SD = 4.41) for problem solving, and 21.09 (SD = 4.29) for avoidance (Table 1). Median values and ranges indicated substantial variability across all scales and subscales.

Pearson correlation analyses revealed associations among alexithymia, difficulties in emotion regulation, coping strategies, and age (Table 2). Total alexithymia scores were moderately and positively correlated with difficulties in emotion regulation (r = 0.49, 95% CI [0.45, 0.53], *p* < 0.001). Higher levels of alexithymia were positively associated with avoidance coping, although the magnitude of this association was small (r = 0.10, 95% CI [0.05, 0.15], *p* < 0.001). Seeking social support and problem solving were positively correlated with each other (r = 0.18, 95% CI [0.12, 0.23], *p* < 0.001). At the subscale level, difficulty identifying feelings and difficulty describing feelings showed moderate to strong associations with both total alexithymia and difficulties in emotion regulation. Externally oriented thinking (EOT) demonstrated a moderate positive correlation with the TAS-20 total score (r = 0.43, 95% CI [0.38, 0.47], *p* < 0.001), but showed negligible associations with difficulties in emotion regulation (r = −0.04, ns). EOT was positively associated with coping strategies, particularly CSI2 (r = 0.21) and CSI3 (r = 0.16).

Age was not significantly associated with total alexithymia or emotion regulation difficulties. However, age showed a small negative association with CSI2 (r = −0.07, *p* < 0.05), suggesting minor developmental variation in specific coping strategies. Female adolescents reported significantly higher levels of alexithymia and greater difficulties in emotion regulation compared to males (Table 3). Emotion regulation difficulties also differed significantly according to academic achievement, with adolescents in the low achievement group exhibiting the highest DERS scores, whereas those with very high achievement reported the lowest levels. Differences in emotion regulation difficulties were also observed according to family type, with adolescents from extended families reporting higher scores compared to those from nuclear families. Post hoc analyses indicated that adolescents with very high academic achievement had significantly lower DERS scores than those in the low achievement group.

Mediation analyses were conducted to examine whether difficulties in emotion regulation statistically mediated the association between alexithymia and coping strategies (Table 4). Across all models, higher levels of alexithymia were strongly associated with greater difficulties in emotion regulation (path a; all *p* < 0.001). Difficulties in emotion regulation were, in turn, significantly associated with all coping outcomes (path b; all *p* < 0.001). For seeking social support, the total effect of alexithymia was not statistically significant (path c), whereas both the direct effect (path c′) and the indirect effect were significant. The bootstrap confidence interval for the indirect effect did not include zero, indicating a significant indirect association. This pattern is consistent with an inconsistent or competitive mediation pattern, in which the indirect and direct effects operate in opposite directions. For problem solving, both the total and direct effects of alexithymia were significant, and the indirect effect through difficulties in emotion regulation was also significant. The indirect effect operated in the opposite direction to the direct effect, suggesting partial mediation within an inconsistent mediation framework. For avoidance coping, the total and indirect effects were significant, whereas the direct effect became non-significant after inclusion of the mediator. This pattern is consistent with an indirect-only statistical mediation pattern. All models were adjusted for age, gender, academic achievement, and family type. Given the cross-sectional design, these findings should be interpreted as statistical associations rather than evidence of temporal or causal mechanisms.

## 4. Discussion

The present study examined the relationships between alexithymia, difficulties in emotion regulation, and coping strategies in adolescents using a model-based analytical approach. Overall, adolescents reported moderate levels of alexithymia, emotion regulation difficulties, and coping strategies. Alexithymia was strongly associated with greater difficulties in emotion regulation, and emotion regulation difficulties were, in turn, associated with reduced use of adaptive coping strategies. Mediation analyses further indicated that difficulties in emotion regulation functioned as a statistical mediator within the proposed model, linking alexithymia to seeking social support and problem solving, even after controlling for demographic variables. These findings extend previous research by moving beyond bivariate associations and providing a model-based statistical perspective on the associations between emotional traits, emotion regulation difficulties, and coping patterns during adolescence.

### 4.1. Alexithymia and Emotion Regulation Difficulties

In the present study, adolescents’ alexithymia levels were found to be moderate (Table 1). The mean TAS-20 score (M = 55.06) falls within the intermediate range when considered alongside commonly used reference thresholds, where scores ≥ 61 are generally interpreted as indicating high alexithymia and scores ≤ 51 as low alexithymia in non-clinical populations. Although cut-off values may vary somewhat across adolescent samples and cultural contexts, the observed mean suggests that alexithymic traits in this sample are neither markedly elevated nor negligible, but rather consistent with a community-based, non-clinical adolescent population.

Previous research has reported relatively high levels of alexithymia among adolescents, attributing this to factors such as depression [16,17], somatic complaints [18], behavioral problems [19], and socioeconomic difficulties [17]. As the current study was conducted in a non-clinical sample, moderate alexithymia levels appear consistent with expectations for a community-based adolescent population.

Similarly, adolescents’ difficulties in emotion regulation were found to be at a moderate level (Table 1). Prior studies have demonstrated that emotion regulation difficulties are associated with a range of psychopathological outcomes, including depression, suicidal ideation, anxiety [20], Tourette syndrome [21], and eating disorders [22]. Given that participants in the present study did not report diagnosed mental disorders, moderate emotion regulation difficulties may reflect normative developmental challenges rather than clinical dysfunction. Adolescence is characterized by ongoing neurocognitive maturation and heightened emotional reactivity, making some degree of difficulty in regulating emotions developmentally expectable.

### 4.2. Relationships Between Alexithymia, Emotion Regulation, and Coping Strategies

Consistent with previous research, a moderate positive association was observed between alexithymia and difficulties in emotion regulation (Table 2). This finding aligns with prior research suggesting that higher alexithymic traits are associated with greater difficulties in recognizing and regulating emotional experiences [23]. From a conceptual perspective, difficulties in identifying and describing feelings may be related to reduced emotional clarity, which in turn is statistically associated with higher levels of emotion regulation difficulties.

With respect to coping strategies, alexithymia showed a small but statistically significant positive association with avoidance coping (Table 2). This finding is consistent with prior evidence suggesting that alexithymic individuals often employ less functional coping strategies [24]. However, it is important to note that these associations are correlational in nature and do not imply causality.

Emotion regulation difficulties showed a negligible, non-significant zero-order association with avoidance coping (r = 0.004, *p* > 0.05, Table 2). However, in the mediation analyses, emotion regulation difficulties were significantly associated with avoidance when controlling for alexithymia and covariates, indicating that this association becomes apparent within the multivariate context.

At the subscale level, EOT demonstrated a moderate positive correlation with the TAS-20 total score (r = 0.43) but showed a negligible and non-significant association with difficulties in emotion regulation (Table 2). EOT was positively correlated with certain coping strategies, particularly problem solving and avoidance, although these associations were small in magnitude. This pattern suggests that, within this adolescent sample, the overall associations of alexithymia with emotion regulation were primarily driven by the Difficulty Identifying Feelings and Difficulty Describing Feelings components rather than EOT. Accordingly, subscale-level interpretations involving EOT were made cautiously. This pattern also highlights that the TAS-20 total score aggregates conceptually distinct components of alexithymia. While the DIF and DDF subscales primarily reflect difficulties in identifying and verbalizing emotional experiences, EOT reflects a cognitive style characterized by externally oriented attention rather than deficits in emotional awareness per se. Consequently, the stronger associations observed for DIF and DDF may indicate that emotion regulation difficulties are more closely related to deficits in emotional awareness than to externally oriented thinking. This interpretation suggests that the TAS-20 total score may capture partially heterogeneous facets of alexithymia, and that subscale-level analyses can provide additional conceptual clarity when interpreting associations with related psychological constructs.

As shown in Table 2, small positive zero-order correlations were observed between total alexithymia and the adaptive coping subscales of the CSI (seeking social support and problem solving). Although this finding may appear inconsistent with the theoretical expectation that higher alexithymia would be associated with lower adaptive coping, the magnitude of these correlations was small and should therefore be interpreted cautiously. In large samples, small associations may emerge due to shared variance among related psychological constructs or overlapping behavioral tendencies rather than reflecting a meaningful adaptive role of alexithymia.

Notably, zero-order correlations between emotion regulation difficulties and coping outcomes were small or negligible. However, in covariate-adjusted regression models, significant associations emerged. This divergence likely reflects shared variance between alexithymia and emotion regulation difficulties. When emotion regulation difficulties were included in the mediation model, the indirect effects revealed the theoretically expected pattern in which higher alexithymia was associated with lower adaptive coping through greater emotion regulation difficulties. This pattern may reflect a statistical suppression effect, whereby associations that are not apparent in zero-order correlations become observable when shared variance between related variables is accounted for in multivariate models. However, because formal suppression diagnostics were not conducted, this interpretation should be considered tentative.

### 4.3. Mediation Model Findings

From a theoretical perspective, the present findings can be situated within conceptual models proposing that effective coping depends on access to differentiated emotional information and the capacity to regulate affective arousal [25,26,27,28]. Within this framework, difficulties in emotion regulation can be conceptualized as a proximal statistical correlate linking alexithymic traits to coping preferences under stress.

Mediation analyses indicated that difficulties in emotion regulation were statistically implicated in the associations between alexithymia and coping outcomes (Table 4). For seeking social support and problem solving, significant indirect effects were observed, while the direct effects remained significant and operated in the opposite direction. This pattern is consistent with an inconsistent (competitive) mediation framework.

In mediation models, the total effect represents the sum of the direct and indirect effects. When these effects operate in opposite directions, their magnitudes may partially offset each other, resulting in a non-significant total effect despite statistically significant component paths. This pattern is commonly referred to as inconsistent or competitive mediation in the statistical mediation literature.

For avoidance coping, the indirect effect was significant, whereas the direct effect became non-significant after inclusion of the mediator. This pattern is consistent with an indirect-only statistical mediation model.

Importantly, given the cross-sectional design, these findings reflect associational patterns compatible with the proposed model rather than evidence of temporal or causal mechanisms. Regarding avoidance, although it is often linked to poorer long-term adjustment when used rigidly, avoidance may also be situationally adaptive by temporarily reducing distress or allowing disengagement from stressors perceived as uncontrollable. Thus, our findings are interpreted as indicating greater reliance on avoidant coping tendencies within this sample, rather than implying that higher avoidance is inherently pathological.

### 4.4. Developmental and Demographic Considerations

Age was not significantly associated with total alexithymia or emotion regulation difficulties in this sample (Table 2). The correlations between age and these variables were negligible in magnitude, suggesting relative stability of alexithymic traits and emotion regulation difficulties across the narrow age range examined (13–17 years).

With respect to coping strategies, age showed a small negative association with problem solving (r = −0.07, *p* < 0.05), whereas correlations with other coping dimensions were negligible. Although statistically significant, the magnitude of this association was small and should be interpreted cautiously. Given the limited age range of the sample, developmental inferences remain restricted.

Gender differences were also observed, with female adolescents reporting higher levels of alexithymia and greater difficulties in emotion regulation than males (Table 3). While several studies—particularly in adult populations—have reported higher alexithymia levels among males, findings in adolescent samples appear more heterogeneous. Some studies attribute elevated emotional difficulties among girls to gender-related socialization processes and heightened emotional sensitivity [29,30], whereas cross-cultural investigations suggest that patterns of alexithymia may vary depending on sociocultural norms regarding emotional expression [31]. Therefore, the present gender-related findings should be interpreted cautiously and warrant replication in longitudinal and multi-method research designs.

Higher academic achievement was associated with lower levels of emotion regulation difficulties (Table 3), supporting evidence that effective emotional regulation is linked to adaptive functioning in academic and interpersonal domains [32]. Differences by family type were also observed, with adolescents from extended families reporting greater difficulties in emotion regulation. Although possible explanations may include contextual or socioeconomic factors, these variables were not directly assessed in the present study. Accordingly, such interpretations remain speculative and hypothesis-generating.

### 4.5. Limitations

Several limitations should be acknowledged. First, the cross-sectional design precludes causal inferences and limits conclusions regarding temporal ordering among alexithymia, emotion regulation difficulties, and coping strategies. Although mediation analyses were conducted, the findings reflect statistical mediation patterns rather than causal mechanisms, and alternative or reciprocal associations cannot be ruled out. Second, all constructs were assessed using adolescent self-report measures administered within a single session, increasing the possibility of shared method variance and common method bias. The absence of multi-informant or behavioral data limits methodological triangulation and may have inflated observed associations. Third, potentially relevant variables—including clinical symptoms, parenting practices, and contextual stressors—were not assessed, restricting the ability to examine broader explanatory pathways. Additionally, participants were recruited within schools and data were collected in classroom settings, which may introduce clustering at the school and/or classroom level. The primary analyses relied on ordinary least squares regression models that assume independence of observations. If intraclass correlation was present, standard errors may have been underestimated, potentially leading to inflated statistical significance. Because school-level identifiers were not available for multilevel modeling or cluster-robust estimation, this issue could not be formally examined. Therefore, the findings should be interpreted with appropriate caution. Future research employing multilevel or cluster-adjusted analytical approaches is warranted to more accurately model hierarchical sampling structures in school-based data. Finally, the use of a school-based convenience sample from a specific geographic and cultural context may limit representativeness and generalizability beyond similar populations.

### 4.6. Clinical Implications

Despite these limitations, the findings have important practical implications. Emotion regulation difficulties emerged as a central mechanism linking alexithymia to reduced use of adaptive coping strategies. Interventions aimed at enhancing emotional awareness, acceptance, and regulation skills may therefore indirectly strengthen adolescents’ coping capacities. School-based programs delivered in collaboration with school counselors and school health professionals may be particularly effective in promoting emotional competence and adaptive coping during adolescence.

Recent evidence from a systematic review suggests that psychological interventions targeting alexithymia primarily focus on enhancing emotional awareness, emotional expression, and emotion regulation capacities [33]. In this context, the present findings provide empirical support for the clinical relevance of emotion regulation as a key intervention target, suggesting that strengthening regulatory skills may indirectly promote more adaptive coping strategies among adolescents with elevated alexithymic traits. School-based preventive programs incorporating emotion regulation–focused components may therefore represent a promising approach for supporting adolescents’ emotional adjustment and coping capacities.

In addition, preventive efforts may benefit from incorporating brief psychoeducational components for parents, aimed at fostering emotionally validating environments that support adolescents’ development of regulatory skills. From a broader public health perspective, early identification of elevated alexithymic traits and emotion regulation difficulties may allow for timely, low-intensity interventions before less adaptive coping tendencies become consolidated.

### 4.7. Future Research Directions

Future studies should employ longitudinal designs to examine whether changes in alexithymia predict subsequent changes in coping through emotion regulation over time. Experimental and intervention-based research is also needed to test whether improving emotion regulation skills leads to improvements in coping strategies, particularly among adolescents with elevated alexithymia. Additionally, incorporating contextual and clinical variables may provide a more comprehensive understanding of the mechanisms underlying maladaptive coping.

## 5. Conclusions

In conclusion, the present findings are consistent with a mediation pattern in which difficulties in emotion regulation are statistically associated with the link between alexithymia and coping strategies among adolescents. Rather than functioning merely as correlated constructs, alexithymia and emotion regulation difficulties appear to be systematically associated within an explanatory model related to adolescents’ engagement in adaptive coping. These findings highlight the importance of supporting emotion regulation capacities as a key target for promoting healthier psychological adjustment during adolescence. While the findings are consistent with a mediation framework, longitudinal and experimental research is necessary to establish temporal ordering and causal pathways.

## Figures and Tables

**Table 1:** Mean scores of adolescents on the TAS-20, TAS subscales (TAS1, TAS2, TAS3), DERS-16 and CSI subscales (CSI1, CSI2, CSI3) (*n* = 1415).

Scale	Mean ± SD	Median (Minimum–Maximum)
TAS-20 (Total)	55.06 ± 10.21	55.00 (20.00–85.00)
TAS1-Difficulty Identifying Feelings	17.40 ± 6.42	17.00 (7.00–35.00)
TAS2-Difficulty Describing Feelings	13.28 ± 4.09	13.00 (5.00–25.00)
TAS3-Externally Oriented Thinking	21.93 ± 3.66	22.00 (9.00–33.00)
DERS-16 (Total)	41.30 ± 15.31	39.00 (16.00–80.00)
CSI subscales		
CSI1-Seeking Social Support	22.00 ± 5.33	22.00 (11.00–33.00)
CSI2-Problem Solving	20.18 ± 4.41	20.00 (12.00–32.00)
CSI3-Avoidance	21.09 ± 4.29	21.00 (11.00–33.00)

Note. Because TAS-20 subscales consist of different numbers of items, raw subscale scores should not be interpreted as directly comparable in magnitude. Higher scores on the TAS-20 and DERS-16 indicate greater alexithymia and greater difficulties in emotion regulation, respectively. For the CSI, higher scores on Seeking Social Support and Problem Solving reflect more adaptive coping strategies, whereas higher scores on Avoidance indicate greater use of maladaptive coping strategies. DERS-16: Difficulty in Emotion Regulation Scale, TAS: Toronto Alexithymia Scale, CSI: Coping Strategies Indicator.

**Table 2:** Pearson correlations among TAS-20 (total and subscales), DERS-16, CSI subscales, and age (n = 1415).

Variables	TAS-20 (Total) r [95% CI]	TAS1-DIF r [95% CI]	TAS2-DDF r [95% CI]	TAS3-EOT r [95% CI]	DERS-16 r [95% CI]	CSI1 r [95% CI]	CSI2 r [95% CI]	CSI3 r [95% CI]
TAS1-DIF	0.878 [0.865, 0.889] **	—						
TAS2-DDF	0.840 [0.824, 0.855] **	0.672 [0.642, 0.699] **	—					
TAS3-EOT	0.425 [0.382, 0.467] **	0.043 [−0.009, 0.095]	0.143 [0.091, 0.194] **	—				
DERS-16	0.494 [0.454, 0.533] **	0.580 [0.544, 0.613] **	0.407 [0.363, 0.450] **	−0.037 [−0.089, 0.015]	—			
CSI1	0.137 [0.085, 0.187] **	0.036 [−0.016, 0.088]	0.182 [0.131, 0.232] **	0.129 [0.078, 0.180] **	−0.011 [−0.063, 0.041]	—		
CSI2	0.190 [0.140, 0.240] **	0.119 [0.067, 0.170] **	0.124 [0.072, 0.175] **	0.206 [0.155, 0.255] **	0.060 [0.008, 0.112] *	0.175 [0.124, 0.225] **	—	
CSI3	0.102 [0.050, 0.153] **	0.045 [−0.007, 0.097]	0.049 [−0.004, 0.100]	0.163 [0.112, 0.214] **	0.004 [−0.049, 0.056]	0.110 [0.058, 0.161] **	0.303 [0.255, 0.349] **	—
Age	0.022 [−0.031, 0.074]	0.030 [−0.023, 0.082]	−0.006 [−0.058, 0.047]	0.017 [−0.035, 0.069]	0.033 [−0.019, 0.085]	−0.049 [−0.101, 0.003]	−0.069 [−0.120, −0.017] *	−0.049 [−0.101, 0.003]

Note. Values represent Pearson correlation coefficients with 95% confidence intervals (in brackets), estimated using Fisher’s r-to-z transformation. * *p* < 0.05, ** *p* < 0.001. TAS = Toronto Alexithymia Scale; DIF = Difficulty Identifying Feelings; DDF = Difficulty Describing Feelings; EOT = Externally Oriented Thinking; DERS = Difficulties in Emotion Regulation Scale; CSI = Coping Strategies Indicator. Higher scores on CSI1 and CSI2 indicate more adaptive coping, whereas higher scores on CSI3 indicate greater use of avoidance coping.

**Table 3:** Differences in alexithymia and emotion regulation difficulties according to selected demographic characteristics (n = 1415).

Variable	Group	TAS-20 (Mean ± SD)	DERS-16 (Mean ± SD)	Test Statistic (TAS/DERS)	*p* (TAS/DERS)
Gender	Female	57.22 ± 9.76	44.84 ± 15.43	t = 9.84/t = 10.87	<0.001/<0.001
	Male	51.96 ± 10.05	36.19 ± 13.61		
Academic Achievement	Very Low	50.67 ± 16.30	42.00 ± 21.43	F = 11.46/F = 8.46	<0.001/<0.001
	Low	56.61 ± 10.85	48.99 ± 17.56		
	Moderate	53.54 ± 10.55	41.99 ± 15.19		
	High	50.22 ± 10.42	38.80 ± 14.35		
	Very High	48.15 ± 8.46	36.76 ± 15.85		
Family Type	Nuclear	52.31 ± 10.59	40.84 ± 15.15	F = 3.42/F = 4.80	0.033/0.008
	Extended	54.89 ± 10.49	45.53 ± 15.13		
	Fragmented	53.45 ± 10.99	41.31 ± 15.31		

Note. Independent samples *t*-tests were used for gender comparisons. One-way ANOVA was conducted for academic achievement and family type comparisons. Tukey’s HSD post hoc tests were applied when omnibus effects were statistically significant. All means and standard deviations represent subgroup-specific descriptive statistics. TAS-20 = Toronto Alexithymia Scale; DERS-16 = Difficulties in Emotion Regulation Scale.

**Table 4:** Mediation analyses examining the indirect effect of emotion regulation difficulties on the relationship between alexithymia and coping strategies (n = 1415).

Outcome Variable (Y)	Path	B	SE	β	t	*p*	95% Bootstrap CI
Seeking Social Support	TAS → DERS (a)	0.949	0.029	0.66	32.99	<0.001	[0.896, 1.002]
	DERS → Social Support (b)	−0.072	0.009	−0.29	−7.91	<0.001	[−0.091, −0.054]
	TAS → Social Support (c)	−0.010	0.010	−0.027	−0.96	0.337	[−0.029, 0.010]
	TAS → Social Support (c′)	0.058	0.013	0.16	4.48	<0.001	[0.033, 0.084]
	Indirect Effect (a × b)	−0.068	—	−0.21	—	—	[−0.087, −0.051]
Problem Solving	TAS → DERS (a)	0.949	0.029	0.66	32.99	<0.001	[0.896, 1.002]
	DERS → Problem Solving (b)	−0.071	0.009	−0.28	−7.82	<0.001	[−0.090, −0.052]
	TAS → Problem Solving (c)	0.031	0.010	0.086	3.14	0.002	[0.012, 0.051]
	TAS → Problem Solving (c′)	0.099	0.013	0.27	7.57	<0.001	[0.074, 0.124]
	Indirect Effect (a × b)	−0.067	—	−0.19	—	—	[−0.086, −0.049]
Avoidance	TAS → DERS (a)	0.949	0.029	0.66	32.99	<0.001	[0.896, 1.002]
	DERS → Avoidance (b)	−0.076	0.009	−0.32	−8.60	<0.001	[−0.093, −0.058]
	TAS → Avoidance (c)	−0.054	0.009	−0.15	−5.76	<0.001	[−0.072, −0.035]
	TAS → Avoidance (c′)	0.018	0.012	0.05	1.48	0.139	[−0.006, 0.043]
	Indirect Effect (a × b)	−0.072	—	−0.21	—	—	[−0.090, −0.055]

Note: a represents the effect of alexithymia (TAS-20 total score) on difficulties in emotion regulation (DERS-16 total score). b represents the covariate-adjusted effect of difficulties in emotion regulation on coping strategies while controlling for alexithymia (TAS-20) and demographic covariates (age, gender, academic achievement, and family type). c represents the total effect of alexithymia on coping strategies. c′ represents the direct effect of alexithymia on coping strategies after inclusion of the mediator. Indirect effects (a × b) were estimated using 5000 bias-corrected bootstrap samples, and 95% confidence intervals are reported. Zero-order correlations among study variables are presented in Table 2 for comparison. Divergences between zero-order and regression-based associations reflect shared variance between alexithymia and emotion regulation difficulties rather than model misspecification. Standardized coefficients (β) are reported for regression paths (a, b, c, c′). Confidence intervals that do not include zero indicate statistically significant effects.

## Data Availability

The data presented in this study are not publicly available due to ethical and privacy considerations. The dataset includes sensitive psychological data collected from adolescent participants (minors) within a school-based setting. In accordance with the ethics committee approval and informed consent procedures, public data sharing was not permitted. However, the data are available from the corresponding author upon reasonable request and subject to ethical approval and data protection regulations.
